# Psychological and Social Work Factors as Predictors of Mental Distress and Positive Affect: A Prospective, Multilevel Study

**DOI:** 10.1371/journal.pone.0152220

**Published:** 2016-03-24

**Authors:** Live Bakke Finne, Jan Olav Christensen, Stein Knardahl

**Affiliations:** Department of Work Psychology and Physiology, National Institute of Occupational Health, Oslo, Norway; University of Westminster, UNITED KINGDOM

## Abstract

Occupational health research has mainly addressed determinants of negative health effects, typically employing individual-level self-report data. The present study investigated individual- *and* department-level (means of each work unit) effects of psychological/social work factors on *mental distress* and *positive affect*. Employees were recruited from 63 Norwegian organizations, representing a wide variety of job types. A total of 4158 employees, in 918 departments, responded at baseline and at follow-up two years later. Multilevel linear regressions estimated individual- and department-level effects simultaneously, and accounted for clustering of data. Baseline exposures *and* average exposures over time ([T1+T2]/2) were tested. *All* work factors; *decision control*, *role conflict*, *positive challenge*, *support from immediate superior*, *fair leadership*, *predictability during the next month*, *commitment to organization*, *rumors of change*, *human resource primacy*, and *social climate*, were related to mental distress *and* positive affect at the *individual* and *department level*. However, analyses of baseline exposures adjusted for baseline outcome, demonstrated significant associations at the *individual* level only. Baseline “rumors of change” was related to mental distress only and baseline “predictability during the next month” was not a statistical significant predictor of either outcome when adjusted for outcome at baseline. Psychological and social work factors were generally related to mental distress and positive affect in a mirrored way. Impact of exposures seemed most pervasive at the individual level. However, department-level relations were also discovered. Supplementing individual-level measures with aggregated measures may increase understanding of working conditions influence on employees`health and well-being. Organizational improvements focusing on the work factors in the current study should be able to reduce distress *and* enhance positive affect. Furthermore, both targeting individual employees *and* redesigning working conditions at the work unit level seems important.

## Introduction

Psychological and social work factors may influence negative mental states like depression, anxiety, and mental distress (systematic reviews: [1, 2–7]). However, working conditions also contribute to *positive* mental states by providing opportunities for achievement, fulfilment, and friendship. Organizational psychology has a long history of investigating determinants of motivation, job satisfaction, commitment, and productivity (e.g. [8, 9, 10]). Karasek and Theorell [11] proposed that the combination of high levels of demands and control promote employee growth and development. Although it is likely that positive emotions will be less salient if negative emotions occur frequently (or vice versa), positive and negative mental states are not mutually exclusive and may co-occur over time (e.g. throughout a day or week) [12–14]. Thus, mental distress and positive affect may have different determinants. Knowledge on how *specific* aspects of work are related to mental distress and positive affect should provide a practical basis for improving working conditions.

The objective of the current study was to determine if and which psychological and social work factors were prospectively related to mental distress *and/or* positive affect among individuals and departments. In addition to capturing relations at a higher level in the organizations, department-level analysis should attenuate possible reporting biases associated with individual reports. Although recent years have seen increased interest in “positive” outcomes (e.g. “engagement”) in occupational health research [15, 16], to our knowledge few prospective studies have investigated specific psychological and social work factors contributing to “negative” and “positive” *mental* states in the *same* study. Furthermore, while previous studies have almost exclusively investigated how an *individual's perception of exposure* to a work factor influences his or her health/well-being [17], the current study utilized multilevel methodology to estimate also how the levels of work factors *between work units* were related to health/well-being. *Mental distress* was defined and measured as symptoms of anxiety and depression [18, 19] while *positive affect* was operationalized as enjoyment of daily activities, alertness, and hope for the future [20].

Research linking work to negative health effects has been dominated by the demand-control (DC; [21]) and the effort-reward imbalance (ERI; [22]) models. Although pivotal in conceptualizing psychological work factors, the dimensions of these models are rather unspecific (for more on this see [23, 24]). Inefficacy of interventions to reduce depression in workers [25] may in part be due to shortage of knowledge of *specific* work factors to modify. Organizational psychology has showed that specific factors like role expectations, organizational changes, aspects of leadership, and organizational climate relate to “positive” outcomes like job motivation, satisfaction, productivity, and performance [8].

The *Job Demands Resources model* (JD-R) has broadened the scope of what is to be considered psychological work exposures and has incorporated both “negative” and “positive” outcomes. In this framework, work factors have been classified under two general categories, “job demands” and “job resources”, defined by their consequences [15, 16]. That is, “demands” refer to any aspect of work that requires sustained effort and thereby “certain physiological and/or psychological costs” ([15] p. 312). Hence, when labeling a factor a “demand” one may have concluded *a priori* that it is aversive. This promotes a circular reasoning and may disguise possible relations (see also [12]). For instance, time pressure has been found to be positively related to “engagement” [26], and may not be a “demand” as defined in the JD-R framework. The current study tested all included factors as possible predictors of *both* mental distress and positive affect.

Many characteristics of work are shared between individuals within work units and investigating relations at this level may justify inferences to the “work environment” [27]. If a factor truly is located at the group level, aggregation (e.g. by calculating means of each work unit) should attenuate bias due to individual employee reporting tendencies [28–30]. For instance, if employees are asked to report the amount of overtime worked in a department, aggregation would remove bias due to variability in the ability to estimate this figure. However, if employees report their own overtime aggregation may remove not only reporting bias, but also unbiased variability in individual reports in order to reflect a group construct. Hence, individual-level measures and aggregated measures may capture different aspects of work factors and thereby supply important complementary information about the influence of work on employee health and well-being.

Individual-level measures reflect partly idiosyncratic information but are not *inherently* biased (see also [31]). Research questions often pertain to *the* '' psychosocial work environment'', seemingly external to and independent of individual employees, but many psychological work factors are subjective by definition and pertain to the job`s content meaning to the employee. Investigating relationships at this level is important to capture factors located at the individual level due to individual differences in both characteristics of the job and the employee`s personality and states. The content and meaning of a job to an employee can only be perceived, appraised, and reported by that person.

The present study employed multilevel modelling to determine both individual-level *and* group-level effects of a comprehensive set of specific psychological and social work factors on *mental distress* and *positive affect* in a sample including several types of jobs. Moreover, a full-panel data set enabled testing several designs to elucidate which factors show the most robust associations with the outcomes *and* allowed the estimation of exposure over time.

## Methods

### Ethics

The study was approved by the Regional Committees for Medical and Health Research Ethics (REK) in Norway, had a specific permission from the Data Inspectorate of Norway and was conducted in accordance with the World Medical Association Declaration of Helsinki. All study participants provided informed consent and data were de-identified for analyses. When accessing the web-based questionnaire by a personal login code, informed consent had to be confirmed before responding to the questionnaire. This consent procedure was approved by the Data Inspectorate of Norway and REK. Furthermore, the approval from the Data Inspectorate required strict procedures for keeping information confidential, and these procedures were communicated to employees before the survey in order to increase response probability and minimize strategic reporting.

### Design

The study employed a prospective two-wave full-panel design. Average time period from baseline to follow-up was 24 months (range 17–36). The current study is part of the comprehensive project “The new workplace: Work, health, and participation in the new work life” carried out by the National Institute of Occupational Health in Norway. A wide range of work factors and outcomes are assessed in this project, and a two-year follow-up period was considered best to capture the various processes under study. Also, participating companies preferred two years to elapse between measurements. A time-lag of at least two years may be necessary to demonstrate a relationship between stressors at work, irritation, and depressive symptoms [32]. However, paucity of knowledge of pathogenic mechanisms precludes the design of an optimal exposure-outcome measurement interval. Therefore, relations of psychological/social work factors with mental distress and positive affect were tested by two statistical designs: (i) modeling mental distress and positive affect (at T2) as a function of exposures at baseline and (ii) modeling mental distress and positive affect (at T2) as a function of average exposure over time ([T1+T2]/2).

### Procedure and subjects

Subjects were recruited from 63 organizations in Norway. Recruitment was done at the organizational level. The project “The new workplace: Work, health, and participation in the new work life”, which this study is a part of, has collected data over a project period of 12 years. At this point, over 100 Norwegian organizations have participated at least once. Of the organizations that were contacted, some agreed to participate and some did not. Also, some organizations contacted NIOH wanting to participate. Hence, although the sample was relatively diverse, reflecting a wide variety of occupations and types of work, sampling was not random.

Invited subjects were distributed across 1252 departments within the organizations. Average number of employees in departments was 11, ranging from 1 to 159 individuals. Baseline data were collected from November 2004 until May 2011, and follow-up data from September 2006 until June 2013. Of the 63 organizations involved, 30 were public and 33 were private. The organizations included municipalities, an insurance company, public organizations, health institutions, and educational institutions, among others, representing a wide variety of job types (see Table 1).

**Table 1:** Baseline descriptives of respondents to the first survey^a^, respondents to the first and second surveys^b^, and drop-outs from the first to the second survey.

	Invited to the first survey (N = 13836)				Invited to the first and second surveys (N = 10274)							
	Respondents to the first survey (N = 7378)				Respondents to the first and second surveys (N = 4158)				Drop-outs from the first to the second survey (N = 1985)			
	N	%	Mean	SD	N	%	Mean	SD	N	%	Mean	SD
**Sex**	.	.	.	.	.	.	.	.	.	.	.	.
Male	2840	38.5	.	.	1650	39.7	.	.	716	36.1	.	.
Female	4538	61.5	.	.	2508	60.3	.	.	1269	63.9	.	.
Missing data	0	0	.	.	0	0	.	.	0	0	.	.
**Age**	.	.	.	.	.	.	.	.	.	.	.	.
< 30	675	9.1	.	.	266	6.4	.	.	179	9.0	.	.
30–39	1917	26.0	.	.	1037	24.9	.	.	504	25.4	.	.
40–49	2189	29.7	.	.	1315	31.6	.	.	597	30.1	.	.
50–59	1944	26.3	.	.	1216	29.2	.	.	549	27.7	.	.
> 59	653	8.9	.	.	324	7.8	.	.	156	7.9	.	.
Missing data	0	0	.	.	0	0	.	.	0	0	.	.
**Classification of occupation**	.	.	.	.	.	.	.	.	.	.	.	.
Legislators, senior officials, and managers	701	9.5	.	.	474	11.4	.	.	124	6.2	.	.
Professionals	2176	29.5	.	.	1296	31.2	.	.	486	24.5	.	.
Technicians and associate professionals	2381	32.3	.	.	1303	31.3	.	.	696	35.1	.	.
Clerks	583	7.9	.	.	304	7.3	.	.	187	9.4	.	.
Service workers and shop and market sales workers	1213	16.4	.	.	617	14.8	.	.	375	18.9	.	.
Skilled agricultural and fishery workers	2	0.0	.	.	1	0.0	.	.	1	0.1	.	.
Craft and related trades workers	85	1.2	.	.	43	1.0	.	.	24	1.2	.	.
Plant and machine operators and assemblers	11	0.1	.	.	1	0.0	.	.	8	0.4	.	.
Elementary occupations	96	1.3	.	.	53	1.3	.	.	33	1.7	.	.
Armed forces and unspecified	33	0.4	.	.	22	0.5	.	.	11	0.6	.	.
Missing data	97	1.3	.	.	44	1.1	.	.	40	2.0	.	.
**Skill level**	.	.	.	.	.	.	.	.	.	.	.	.
Competence equivalent to minimum 4 years of higher education (> 16 years)	2176	29.5	.	.	1296	31.2	.	.	486	24.5	.	.
Competence equivalent to 1–3 years of higher education (13–15 years)	2381	32.3	.	.	1303	31.3	.	.	696	35.1	.	.
Competence equivalent to high school (10–12 years)	1894	25.7	.	.	966	23.2	.	.	595	30.0	.	.
Occupations that do not require high school (< 10 years)	96	1.3	.	.	53	1.3	.	.	33	1.7	.	.
Occupations with unspecified requirements for competence	734	9.9	.	.	496	11.9	.	.	135	6.8	.	.
Missing data	97	1.3	.	.	44	1.1	.	.	40	2.0	.	.
**Mental distress**	.	.	.	.	.	.	.	.	.	.	.	.
Mean score	.	.	1.38	0.41	.	.	1.37	0.40	.	.	1.39	0.40
Missing data	.	.	181	181	.	.	98	98	.	.	66	66
**Positive affect**	.	.	.	.	.	.	.	.	.	.	.	.
Mean score	.	.	3.97	0.78	.	.	3.99	0.77	.	.	3.94	0.78
Missing data	.	.	790	790	.	.	425	425	.	.	223	223

^a^Respondents were defined as having completed the HSCL-10 and/or the three WAI items, minimum one predictor at the first survey, and having information on department affiliation.

^b^Respondents were defined as having completed the HSCL-10 and/or the three WAI items at both the first and second surveys, minimum one predictor at the first survey, and having information on department

All data were measured at the *individual* level, within organizations. The questionnaire gathered data on background, a wide range of physical, psychological and organizational factors, and both mental and somatic health complaints. This study is based on parts of this information. Organizations received written reports and oral presentations of results of the work environment survey as a tool for organizational development and an aid for monitoring the organizational work environment.

Information regarding the project was given to employees and management through oral presentations at the organizational level. The organizations supplied lists containing names, addresses, sex, age, personal identification numbers, departmental affiliation, and classification of the occupations of all their employees. Letters with information of the purpose of the study and confidentiality, and either a personal access code to the web-based questionnaire or a paper version of the questionnaire were mailed to all employees. For further details about the data collection procedure, see [24].

Occupation was classified according to the standard classification of occupations (STYRK), developed by Statistics Norway (www.ssb.no) based on the International Standard Classification of Occupation (ISCO-88). One criterion for this classification is technical and formal skills normally required for a certain occupation. The classification is not based on obtained formal education, but reflects the education level normally required by a given occupation.

A total of 10274 employees were invited at both baseline and follow-up. Of these, 4158 (40.5%) were included as respondents (Table 1). Response was defined as having completed at least one psychological/social work factor at T1 and one of the outcome measures (i.e. Hopkins Symptom Checklist (HSCL-10) and/or “Mental Resources” of the Work Ability Index (WAI)) at *both* T1 and T2. Subjects were excluded if information about departmental affiliation lacked, as this information was necessary to conduct multilevel analyses. Thus, in the final sample respondents were distributed across 918 departments within 63 organizations with an average number of 5 (range 1–35) individuals in each department.

### Measures

#### Mental distress

Degree of mental distress (symptoms of anxiety and depression) *during the last week* was measured by a Norwegian translation of the Hopkins Symptom Checklist-10 (HSCL-10). HSCL has shown adequate psychometric properties [19] and is a frequently used self-report instrument to assess mental distress in population surveys [18]. The different versions of the instrument range from five to 90 items [33]. HSCL-10 is an abbreviated version of HSCL-25. Correlation between these instruments was 0.97 in a previous validation study [34]. Examples of items in HSCL-10 are “feeling tense or keyed up” and “feeling hopeless about the future”. Responses are given on a four-point scale: 1 = “Not at all”, 2 = “A little”, 3 = “Quite a bit”, and 4 = “Extremely”. Missing values were replaced with the individual mean, but responders with three or more missing items were excluded. This constituted 6 (0.1%) responders at T1 and 12 (0.3%) at T2. Cronbach’s α was 0.86 at T1 and 0.87 at T2.

#### Positive affect

Three items (translated into Norwegian) from the Work Ability Index (WAI) [20] measured degree of “positive affect” (in WAI labelled “Mental Resources”). WAI assesses the ability of an employee to perform their job, taking into account the demands (physical and mental) of work, the worker's health status, and resources [35]. The instrument has been widely applied in scientific studies of occupational health and also in clinical practice [36, 37], and adequate psychometric properties have been demonstrated (e.g. [38, 39]). The instrument consists of seven dimensions, each included in an index ranging 7–49 and classified into poor (7–27), moderate (28–36), good (37–43), and excellent (44–49) work ability [20]. In the current study, only the dimension measuring “mental resources” was employed. “Mental resources” is composed of three items: “Have you been able to enjoy your regular daily activities recently”, “have you been active and alert recently”, and “have you felt yourself to be full of hope for the future recently”. Responses are given on a five-point frequency scale: 1 = “never”, 2 = “rather seldom”, 3 = “sometimes”, 4 = “rather often”, and 5 = “often”. The variable was studied as continuous. Cronbach’s α was 0.85 at T1 and 0.86 at T2.

#### Psychological trait variables: Optimism

Dispositional optimism was measured by three items from the Life Orientation Test (LOT) [40, 41]: “In uncertain times, I usually expect the best”, “I hardly ever expect things to go my way”, and “overall, I expect more good things to happen to me than bad”. The response scale was: “1 = strongly disagree”, “2 = disagree”, “3 = neutral”, “4 = agree”, and “5 = strongly agree”. The original LOT consists of 12 items [40], and there is also a revised version (LOT-R) [41] consisting of 10 items that is widely used. Cronbach’s α of the three current items was 0.61 at T1 and 0.60 at T2.

#### Psychological and social work factors

Psychological and social work factors were assessed with the General Nordic Questionnaire for Psychological and Social Factors at Work (QPS_Nordic_) [8]. QPS_Nordic_ is a validated instrument for research and also a tool for monitoring and improving working conditions in organizations [8, 42]. Scales included in the current study were; *quantitative demands* (time pressure and amount of work), *decision control* (influence on decisions regarding work tasks, choice of coworkers, and contacts with clients), *role conflict* (conflicts between demands and resources, conflicting requests, illegitimate tasks), *support from immediate superior* (instrumental and emotional support, and appreciation), *empowering leadership* (encouragement of participation in important decisions and expressing differing opinions, development of skills), *fair leadership* (fairness of task distribution and fair and equal treatment of employees), *predictability during the next month* (predictability of tasks, coworkers, and superiors), *commitment to organization* (positive feelings and attitudes towards the workplace), *social climate* (whether the social climate is encouraging/supportive, distrustful/suspicious, relaxed/comfortable), *positive challenge at work* (usefulness of skills and knowledge, meaningfulness of work, and whether work is challenging in a positive way), and *human resource primacy* (organizational practices pertaining to reward for well executed job tasks, taking care of employees, the interest of management in the health and well-being of employees). The scales varied from three to five items. Cronbach’s alphas ranged from 0.62 to 0.91 at baseline and from 0.64 to 0.92 at follow-up.

The response scale was: “1 = very seldom or never”, “2 = somewhat seldom”, “3 = sometimes”, “4 = somewhat often”, and “5 = very often or always”. Exceptions were *commitment to organization* with the response alternatives: “1 = disagree totally”, “2 = disagree to some extent”, “3 = indifferent”, “4 = agree to some extent”, and “5 = agree totally”, and *human resource primacy* and *social climate*: “1 = very little or not at all”, “2 = rather little”, “3 = somewhat”, “4 = rather much”, and “5 = very much”.

A *single item* from QPS_Nordic_ was also included. “Are there rumors concerning changes at your workplace?” with the response scale “1 = very seldom or never” to “5 = very often or always”.

A single item measured *organizational procedural justice* [43] related to organizational change: “Procedures are designed to hear the concerns of all those affected by the decision” with the response alternatives “1 = strongly agree”, “2 = quite agree”, “3 = neutral”, “4 = quite disagree”, and “5 = strongly disagree”.

### Statistical analyses

Statistical analyses were conducted with SPSS Statistics, version 19.0 (IBM, Armonk, NY, USA), Mplus Version 6.11 [44], and R Version 3.0.2 [45].

The association of sex and age with *non-response* was estimated with univariable logistic regression analyses. All individuals invited at baseline were included in the analyses.

*Attrition bias* was tested with logistic regressions. For baseline responders, the odds of *also* responding at follow-up were computed. Predictors in univariable regressions were age, sex, skill level, mental distress (T1), positive affect (T1), and psychological/social work factors. Statistically significant predictors were subsequently entered in a multivariable regression.

Statistical analyses to estimate relations between work factors and the two outcomes mental distress and positive affect were conducted in two steps; First, individual-level ordinary least square (OLS) regressions were run with a *comprehensive* set of work factors. Then, based on results of OLS regressions some factors were chosen for more extensive scrutiny by multilevel linear regression analyses comprising both the individual- and group level. Aggregated scores at the group level were obtained by calculating means of each department.

In the first step a broad set of work factors were tested in separate OLS regressions with baseline exposures. To adjust for possible confounding, age, sex, skill level, and the outcome at baseline were included as covariates in all analyses. In addition, dispositional optimism was included to test and adjust for possible confounding by personality-contingent reporting bias.

Multilevel modelling (MLM) has several advantages over more conventional methods such as OLS regression. Firstly, contrary to OLS, MLM accounts for possible lack of independence of observations within clusters (e.g. departments), which may affect estimates (e.g. deflate standard errors and increase type I error) [46, 47]. Secondly, a basic two-level MLM allows for the simultaneous estimation of regression coefficients *within* each level 2 unit (i.e. “within-level” effects) using individual-level scores, and effects *between* level 2 units (i.e. “between-level” effects) using departmental means. *Random effects* (i.e. random *intercepts* and random *slopes*) are utilized to model variability of regression parameters between departments. In a *random intercept only* model variation in the outcome variable between departments is accounted for by allowing intercepts of the individual level regressions to vary between departments. In a *random intercept and slope* model regression *slopes* are also allowed to vary between departments, meaning that both unexplained variance (intercepts) and variance explained by predictors (slopes) are allowed to vary between departments [47].

The participants of the current study were clustered in organizations and departments. Departmental affiliation was used as cluster variable as employees seemed more likely to be influenced by shared conditions within departments than at the organizational level (for more on this see [23]). Intraclass-correlations (ICC1s) were estimated to investigate how much of the total variance in the work factors could be attributed to between-level differences (ICC1) [47, 48]. Furthermore, “null-models” with outcome variables only were estimated for each outcome to examine whether departments statistically significantly differed from each other in average levels of distress and positive affect.

As work factors at the aggregated level may be considered *reflective* measures with the individual-level responses as interchangeable indicators of the higher level construct (see e.g. [49, 50]), work factors were group-mean centred in multilevel models; the departmental mean of the work factors was subtracted from each individual`s score. Adjusting the individual-level ratings to the respective cluster mean provides a way of disentangling group effects from individual effects—the individual level predictor refers to each individual`s relative position to their group mean [50]. It should be noted that between-group effects of the aggregated variables are thus modeled *as if* independent of, rather than adjusted for, individual-level effects. Hence, although the influence of idiosyncracies of individual employees should be attenuated by department-level aggregation, group effects are not pure “contextual effects” that are purged of inter-individual differences. This approach was chosen since for most work factors the group-level construct must be considered inextricably linked with employee perceptions. Partialing out inter-individual differences completely may remove the phenomenon of interest, insofar as the effect of a group factor is mediated by an individual psychological response (for more on group-mean centering, see [50]). To adjust for age, sex, skill level, and the outcome parameter at baseline at *both* levels these variables were grand-mean centred—i.e. the overall mean was subtracted from each individual`s score [50]. Both random intercept and random slope models were tested. The Bayesian information criterion (BIC) was employed to decide whether intercept only or intercept and slope models should be preferred. The model with the lowest BIC value is the better fitting model [51].

Previous prospective studies have mostly examined effects of exposure measured at one time point. Due to limited knowledge of what time interval should be applied when studying health effects of different work factors [52] and that exposure may fluctuate over time, it has been recommended to include more than one single assessment of exposure [2, 5–7]. Designating exposure based on one time point only may constitute misclassification (for more on this see [23]). Hence, multilevel analyses estimated the effect of levels of exposure both at (A) baseline and (B) averaged over time ([T1+T2]/2). The baseline model estimated possible long-term *effects* while the average model estimated effects of long-term *exposure*.

To reduce the risk of type I error when conducting multiple tests, 99% confidence intervals were employed. Age, sex, skill level, and the outcome (mental distress/positive affect) at baseline were included in all multivariable analyses. The practice of baseline adjustment has been debated, and may constitute severe over adjustment (e.g. [53, 54]). Hence, we also ran models without baseline adjustment for the outcome.

As the focus of the current study was to explore how the included work factors were related to mental distress and positive affect applying the extensive statistical approach of multilevel modelling, each work factor was modelled separately. Mutually adjusting for all other exposures in this comprehensive study would diminish statistical power and constitute over adjustment. This is particularly inappropriate if the included factors are causally related in other ways, for instance by mediating the effects of each other. Statistical procedures alone cannot distinguish between mediation and confounding [55]. Previous research identifying confounders is to our knowledge lacking, and “blindly” entering control variables into models may severely bias effects [7].

## Results

Among respondents at baseline the three largest occupational groups were *technicians and associate professionals* (N = 2381, 32.3%), *professionals* (N = 2176, 29.5%), and *service workers and shop and market sales workers* (N = 1213, 16.4%) (Table 1). For those responding at both baseline and follow-up the corresponding figures were 31.3% (N = 1303), 31.2 (N = 1296), and 14.8% (N = 617).

Baseline responders exhibited a mean *mental distress* score of 1.38 (SD 0.41) and a *positive affect* score of 3.97 (SD 0.78) (Table 1). The cut-off score defining clinically relevant distress in a Norwegian population is 1.85 [34]. For responders at both baseline and follow-up, mean mental distress score was 1.37 (SD 0.40), and mean positive affect score was 3.99 (SD 0.77). In the prospective sample there was a statistically significant correlation between mental distress and positive affect at both T1 (r = -0.51, p< .000) and T2 (r = -0.53, p< .000). Furthermore, the correlation between mental distress at T1 and mental distress at T2 was 0.67 (p< .000), and between positive affect at T1 and T2 it was 0.54 (p< .000).

**Non-response** analysis demonstrated that age groups 30–39, 40–49, and 50–59 displayed statistically significantly increased odds of responding compared to the lowest age group (< 30) (Table 2). Sex did not predict responding.

**Table 2:** Separate univariable logistic regression analyses to estimate non-response at baseline and attrition from baseline to follow-up.

	Non-response analyses^a^			Attrition analyses^b^		
	N	OR	95% CI	N	OR	95% CI
**Sex**	.	.	.	.	.	.
Male	5098	1.00	ref	2366	1.00	ref
Female	8218	0.98	0.91–1.05	3777	**0.86**	0.77–0.96*
**Age**	.	.	.	.	.	.
< 30	1397	1.00	ref	445	1.00	ref
30–39	3353	**1.43**	1.26–1.62*	1541	**1.39**	1.11–1.72*
40–49	3814	**1.44**	1.27–1.63*	1912	**1.48**	1.20–1.83*
50–59	3410	**1.42**	1.25–1.61*	1765	**1.49**	1.20–1.85*
> 59	1335	1.02	0.88–1.19	480	**1.40**	1.07–1.83*
**Skill level**	.	.	.	.	.	.
Competence equivalent to minimum 4 years of higher education (> 16 years) (> 16 years)	.	.	.	1782	1.00	ref
Competence equivalent to 1–3 years of higher education (13–15 years) (13–15 years)	.	.	.	1999	**0.70**	0.61–0.81*
Competence equivalent to high school (10–12 years)	.	.	.	1561	**0.61**	0.53–0.70*
Occupations that do not require high school (< 10 years)	.	.	.	86	**0.60**	0.39–0.94*
Occupations with unspecified requirements for competence	.	.	.	631	**1.38**	1.11–1.71*
**Mental distress**	.	.	.	5979	**0.86**	0.75–0.98*
**Positive affect**	.	.	.	5495	**1.08**	1.01–1.16*

^a^Respondents were defined as having completed the HSCL-10 and/or the three WAI items, minimum one predictor at the first survey, and having information on department affiliation.

^b^Respondents were defined as having completed the HSCL-10 and/or the three WAI items at both the first and second surveys, minimum one predictor at the first survey, and having information on department affiliation at the first survey.

*p < .05

**Attrition** analysis exhibited that females were less likely to drop out after baseline. All age groups (30–39, 40–49, 50–59, and > 59) were associated with higher odds of responding than the youngest (< 30). Employees in occupations requiring the equivalent of > 16 years of education exhibited higher odds of responding than the groups requiring 13–15, 10–12, and < 10 years of education, and lower odds of responding than those in occupations with unspecified requirements for competence. Among respondents at baseline, a higher level of mental distress predicted decreased odds of responding at follow-up while reporting higher scores on positive affect were associated with increased odds of responding two years later (Table 2).

Multivariable attrition analysis demonstrated that *skill level* and *social climate* were statistically significant predictors of responding at follow-up (analysis not shown). The groups with requirements of competence equivalent of 10–12 years (odds ratio [OR] 0.63, 95% confidence interval [CI]: 0.53–0.76) and 13–15 years (OR 0.69, 95% CI: 0.58–0.82) of education displayed lowered odds of responding. Higher scores on social climate were associated with increased odds of responding at follow-up (OR 1.14, 95% CI: 1.02–1.29).

Based on results from **individual-level OLS regression** analyses (see S1 and S2 Tables, available through hyperlink) the following 10 work factors were chosen to be included in multilevel models; *decision control*, *role conflict*, *positive challenge*, *support from immediate superior*, *fair leadership*, *predictability during the next month*, *commitment to the organization*, *rumors of change*, *human resource primacy*, and *social climate*. These factors reflect evaluations of both the task, social relations, and perceptions of the organizational. Eight of the factors were statistically significantly related to *both* mental distress and positive affect (i.e. decision control, role conflict, positive challenge, support from immediate superior, fair leadership, human resource primacy, and social climate) while rumors of change was related to mental distress only and predictability during the next month and commitment to the organization were predictors of positive affect only (see S1 and S2 Tables, available through hyperlink).

**Intraclass-correlations** (ICC(1)s) for *baseline exposures* and *average exposures* were; *decision control* (0.17 and 0.21), *role conflict* (0.11 and 0.14), *support from immediate superior* (0.14 and 0.14), *fair leadership* (0.15 and 0.16), *predictability during the next month* (0.15 and 0.17), *rumors of change* (0.30 and 0.35), *commitment to organization* (0.26 and 0.26), *positive challenge* (0.15 and 0.19), *human resource primacy* (0.28 and 0.29), and *social climate* (0.18 and 0.21). All coefficients were above the recommended level of 0.05 [48] indicating sufficient between-groups variation to justify departmental level aggregation.

Between-groups variation for the outcomes *mental distress* and *positive affect* is presented in Table 3.

**Table 3:** Multilevel linear regression”null models” with outcome variables mental distress and positive affect.

	Null model							
	Mental distress				Positive affect			
	N	Var.comp	*SE*	P-value	N	Var.comp	*SE*	P-value
	5316	.	.	.	4663	.	.	.
**Individual level intercept variance**	.	**0.169**	**0.006**	**0.000**	.	**0.704**	**0.015**	**0.000**
**Department level intercept variance**	.	**0.004**	**0.002**	**0.014**	.	**0.030**	**0.007**	**0.000**

### Multilevel linear regressions

Applying multilevel analyses to a wide range of work factors for two separate outcomes necessitated a vast number of regression analyses. Thus, only “fixed effects” (i.e. beta-coefficients) will be reported here. “Random components” (i.e. intercept residual variance at the individual level and department level, and variance of slopes) can be found in S3 and S4 Tables, available through hyperlink. Furthermore, whether *random intercept only models* or *random intercept and slope models* exhibited best fit to the data [51] will not be reported here, but indicated in table notes only (Tables 4 and 5). Results for each outcome will be presented separately in text and tables (see Tables 4 and 5). Additionally, a table presenting a summary of fixed effects across outcomes is included (see Table 6).

**Table 4:** Fixed effects from multilevel linear regression models with psychological and social work factors at baseline and averaged across time ([T1+T2]/2) as predictors of mental distress at follow-up^a^.

Exposure	Baseline exposure as predictor								Average exposure as predictor^c^			
	No adjustment for baseline mental distress^b^				Adjusted for baseline mental distress^c^							
	N	B	*SE*	P-value	N	B	*SE*	P-value	N	B	*SE*	P-value
**Decision control**	4262^e^	.	.	.	3978^d^	.	.	.	3966^e^	.	.	.
Intercept	.	**1.595**	**0.052**	**0.000**	.	**1.420**	**0.039**	**0.000**	.	**1.498**	**0.041**	**0.000**
Individual level	.	**-0.102**	**0.012**	**0.000**	.	**-0.029**	**0.009**	**0.001**	.	**-0.067**	**0.011**	**0.000**
Department level	.	**-0.072**	**0.016**	**0.000**	.	-0.015	0.01	0.224	.	**-0.040**	**0.013**	**0.002**
**Role conflict**	4281^d^	.	.	.	3991^d^	.	.	.	3982^d^	.	.	.
Intercept	.	**1.161**	**0.045**	**0.000**	.	**1.322**	**0.034**	**0.000**	.	**1.232**	**0.032**	**0.000**
Individual level	.	**0.135**	**0.010**	**0.000**	.	**0.042**	**0.008**	**0.000**	.	**0.089**	**0.010**	**0.000**
Department level	.	**0.086**	**0.018**	**0.000**	.	0.021	0.014	0.121	.	**0.058**	**0.013**	**0.000**
**Positive challenge**	4086^e^	.	.	.	3815^e^	.	.	.	3683^e^	.	.	.
Intercept	.	**1.785**	**0.076**	**0.000**	.	**1.479**	**0.060**	**0.000**	.	**1.577**	**0.062**	**0.000**
Individual level	.	**-0.116**	**0.013**	**0.000**	.	**-0.039**	**0.010**	**0.000**	.	**-0.080**	**0.011**	**0.000**
Department level	.	**-0.103**	**0.018**	**0.000**	.	-0.026	0.015	0.070	.	**-0.050**	**0.015**	**0.001**
**Support from immediate superior**	4266^e^	.	.	.	3997^e^	.	.	.	3985^e^	.	.	.
Intercept	.	**1.654**	**0.061**	**0.000**	.	**1.402**	**0.048**	**0.000**	.	**1.587**	**0.050**	**0.000**
Individual level	.	**-0.128**	**0.010**	**0.000**	.	**-0.043**	**0.008**	**0.000**	.	**-0.085**	**0.009**	**0.000**
Department level	.	**-0.073**	**0.015**	**0.000**	.	-0.007	0.012	0.551	.	**-0.055**	**0.013**	**0.000**
**Fair leadership**	4225^e^	.	.	.	3970^e^	.	.	.	3947^e^	.	.	.
Intercept	.	**1.691**	**0.066**	**0.000**	.	**1.409**	**0.050**	**0.000**	.	**1.630**	**0.051**	**0.000**
Individual level	.	**-0.131**	**0.011**	**0.000**	.	**-0.038**	**0.009**	**0.000**	.	**-0.095**	**0.010**	**0.000**
Department level	.	**-0.081**	**0.016**	**0.000**	.	-0.009	0.012	0.486	.	**-0.065**	**0.013**	**0.000**
**Predictability during the next month**	4283^d^	.	.	.	3999^d^	.	.	.	3984^e^	.	.	.
Intercept	.	**1.663**	**0.077**	**0.000**	.	**1.412**	**0.060**	**0.000**	.	**1.586**	**0.067**	**0.000**
Individual level	.	**-0.063**	**0.012**	**0.000**	.	-0.008	0.009	0.354	.	**-0.047**	**0.012**	**0.000**
Department level	.	**-0.069**	**0.018**	**0.000**	.	-0.009	0.014	0.534	.	**-0.050**	**0.016**	**0.001**
**Commitment to organization**	4117^e^	.	.	.	3901^e^	.	.	.	3889^e^	.	.	.
Intercept	.	**1.586**	**0.048**	**0.000**	.	**1.360**	**0.037**	**0.000**	.	**1.509**	**0.039**	**0.000**
Individual level	.	**-0.105**	**0.010**	**0.000**	.	**-0.022**	**0.008**	**0.008**	.	**-0.074**	**0.009**	**0.000**
Department level	.	**-0.057**	**0.013**	**0.000**	.	0.004	0.010	0.695	.	**-0.035**	**0.010**	**0.000**
**Rumors of change**	4244^e^	.	.	.	3962^d^	.	.	.	3924^d^	.	.	.
Intercept	.	**1.252**	**0.026**	**0.000**	.	**1.363**	**0.021**	**0.000**	.	**1.302**	**0.022**	**0.000**
Individual level	.	**0.077**	**0.008**	**0.000**	.	**0.021**	**0.006**	**0.000**	.	**0.052**	**0.007**	**0.000**
Department level	.	**0.041**	**0.009**	**0.000**	.	0.004	0.007	0.563	.	**0.026**	**0.008**	**0.001**
**Human resource primacy**	4041^d^	.	.	.	3833^d^	.	.	.	3706^e^	.	.	.
Intercept	.	**1.545**	**0.039**	**0.000**	.	**1.364**	**0.031**	**0.000**	.	**1.470**	**0.032**	**0.000**
Individual level	.	**-0.137**	**0.010**	**0.000**	.	**-0.043**	**0.008**	**0.000**	.	**-0.086**	**0.010**	**0.000**
Department level	.	**-0.056**	**0.012**	**0.000**	.	0.003	0.010	0.750	.	**-0.031**	**0.010**	**0.002**
**Social climate**	4216^e^	.	.	.	3964^e^	.	.	.	3922^e^	.	.	.
Intercept	.	**1.770**	**0.064**	**0.000**	.	**1.463**	**0.053**	**0.000**	.	**1.657**	**0.052**	**0.000**
Individual level	.	**-0.152**	**0.013**	**0.000**	.	**-0.037**	**0.010**	**0.000**	.	**-0.103**	**0.011**	**0.000**
Department level	.	**-0.104**	**0.017**	**0.000**	.	-0.023	0.014	0.089	.	**-0.074**	**0.013**	**0.000**

^a^Separate regressions were run for each factor.

^b^Age, sex, and skill level were included in all regressions.

^c^Age, sex, skill level, and mental distress at baseline (T1) were included in all regressions.

^d^Random intercept only model

^e^Random intercept and slope model

**Table 5:** Fixed effects from multilevel linear regression models with psychological and social work factors at baseline and averaged across time ([T1+T2]/2) as predictors of positive affect at follow-up^a^.

Exposure	Baseline exposure as predictor								Average exposure as predictor^c^			
	No adjustment for baseline positive affect^b^				Adjusted for baseline positive affect^c^							
	N	B	*SE*	P-value	N	B	*SE*	P-value	N	B	*SE*	P-value
**Decision control**	3768^d^	.	.	.	3206^d^	.	.	.	3199^d^	.	.	.
Intercept	.	**3.484**	**0.118**	**0.000**	.	**3.789**	**0.106**	**0.000**	.	**3.573**	**0.107**	**0.000**
Individual level	.	**0.204**	**0.023**	**0.000**	.	**0.083**	**0.021**	**0.000**	.	**0.142**	**0.026**	**0.000**
Department level	.	**0.134**	**0.038**	**0.000**	.	0.036	0.035	0.302	.	**0.105**	**0.034**	**0.002**
**Role conflict**	3786^d^	.	.	.	3216^d^	.	.	.	3209^d^	.	.	.
Intercept	.	**4.214**	**0.104**	**0.000**	.	**4.023**	**0.088**	**0.000**	.	**4.147**	**0.084**	**0.000**
Individual level	.	**-0.206**	**0.022**	**0.000**	.	**-0.088**	**0.022**	**0.000**	.	**-0.145**	**0.025**	**0.000**
Department level	.	**-0.130**	**0.041**	**0.002**	.	-0.050	0.035	0.154	.	**-0.101**	**0.034**	**0.003**
**Positive challenge**	3618^d^	.	.	.	3092^d^	.	.	.	3015^d^	.	.	.
Intercept	.	**3.034**	**0.164**	**0.000**	.	**3.545**	**0.147**	**0.000**	.	**3.135**	**0.155**	**0.000**
Individual level	.	**0.292**	**0.025**	**0.000**	.	**0.150**	**0.024**	**0.000**	.	**0.235**	**0.028**	**0.000**
Department level	.	**0.214**	**0.040**	**0.000**	.	0.088	0.036	0.014	.	**0.187**	**0.038**	**0.000**
**Support from immediate superior**	3777^d^	.	.	.	3222^d^	.	.	.	3211^d^	.	.	.
Intercept	.	**3.202**	**0.127**	**0.000**	.	**3.696**	**0.111**	**0.000**	.	**3.301**	**0.118**	**0.000**
Individual level	.	**0.216**	**0.018**	**0.000**	.	**0.091**	**0.018**	**0.000**	.	**0.176**	**0.021**	**0.000**
Department level	.	**0.179**	**0.032**	**0.000**	.	0.052	0.028	0.065	.	**0.153**	**0.030**	**0.000**
**Fair leadership**	3737^d^	.	.	.	3201^e^	.	.	.	3187^d^	.	.	.
Intercept	.	**3.335**	**0.142**	**0.000**	.	**3.757**	**0.129**	**0.000**	.	**3.290**	**0.131**	**0.000**
Individual level	.	**0.213**	**0.021**	**0.000**	.	**0.093**	**0.021**	**0.000**	.	**0.197**	**0.024**	**0.000**
Department level	.	**0.142**	**0.036**	**0.000**	.	0.036	0.032	0.260	.	**0.153**	**0.032**	**0.000**
**Predictability during the next month**	3787^d^	.	.	.	3222^d^	.	.	.	3208^d^	.	.	.
Intercept	.	**3.262**	**0.175**	**0.000**	.	**3.577**	**0.160**	**0.000**	.	**3.190**	**0.168**	**0.000**
Individual level	.	**0.102**	**0.025**	**0.000**	.	0.057	0.025	0.025	.	**0.111**	**0.030**	**0.000**
Department level	.	**0.149**	**0.041**	**0.000**	.	0.076	0.038	0.042	.	**0.166**	**0.039**	**0.000**
**Commitment to organization**	3669^e^	.	.	.	3165^d^	.	.	.	3157^d^	.	.	.
Intercept	.	**3.381**	**0.106**	**0.000**	.	**3.825**	**0.100**	**0.000**	.	**3.528**	**0.104**	**0.000**
Individual level	.	**0.221**	**0.021**	**0.000**	.	**0.075**	**0.019**	**0.000**	.	**0.167**	**0.022**	**0.000**
Department level	.	**0.136**	**0.028**	**0.000**	.	0.019	0.026	0.467	.	**0.097**	**0.027**	**0.000**
**Rumors of change**	3757^d^	.	.	.	3201^d^	.	.	.	3174^d^	.	.	.
Intercept	.	**4.086**	**0.060**	**0.000**	.	**3.968**	**0.054**	**0.000**	.	**4.057**	**0.054**	**0.000**
Individual level	.	**-0.110**	**0.017**	**0.000**	.	-0.026	0.016	0.115	.	**-0.064**	**0.020**	**0.001**
Department level	.	**-0.065**	**0.020**	**0.001**	.	-0.024	0.018	0.194	.	**-0.057**	**0.019**	**0.003**
**Human resource primacy**	3589^d^	.	.	.	3116^d^	.	.	.	3042^d^	.	.	.
Intercept	.	**3.509**	**0.087**	**0.000**	.	**3.842**	**0.079**	**0.000**	.	**3.552**	**0.083**	**0.000**
Individual level	.	**0.261**	**0.022**	**0.000**	.	**0.106**	**0.021**	**0.000**	.	**0.188**	**0.024**	**0.000**
Department level	.	**0.123**	**0.027**	**0.000**	.	0.018	0.025	0.472	.	**0.107**	**0.026**	**0.000**
**Social climate**	3732^d^	.	.	.	3197^d^	.	.	.	3166^d^	.	.	.
Intercept	.	**3.117**	**0.145**	**0.000**	.	**3.645**	**0.133**	**0.000**	.	**3.235**	**0.140**	**0.000**
Individual level	.	**0.246**	**0.023**	**0.000**	.	**0.075**	**0.023**	**0.001**	.	**0.193**	**0.026**	**0.000**
Department level	.	**0.204**	**0.038**	**0.000**	.	0.067	0.034	0.052	.	**0.173**	**0.036**	**0.000**

^a^Separate regressions were run for each factor.

^b^Age, sex, and skill level were included in all regressions.

^c^Age, sex, skill level, and positive affect at baseline (T1) were included in all regressions.

^d^Random intercept only model

^e^Random intercept and slope model

**Table 6:** Summary of fixed effects from multilevel linear regression models across outcomes (mental distress and positive affect) ^a^. “+” signifies a statistical significant effect, and “**÷**” signifies no statistical significant effect.

Exposure	Baseline exposure as predictor				Average exposure as predictor^c^	
	No adjustment for baseline outcome^b^		Adjusted for baseline outcome^c^			
	Mental distress	Positive affect	Mental distress	Positive affect	Mental distress	Positive affect
**Decision control**	.	.	.	.	.	.
Individual level	**+**	**+**	**+**	**+**	**+**	**+**
Department level	**+**	**+**	**÷**	**÷**	**+**	**+**
**Role conflict**	.	.	.	.	.	.
Individual level	**+**	**+**	**+**	**+**	**+**	**+**
Department level	**+**	**+**	**÷**	**÷**	**+**	**+**
**Positive challenge**	.	.	.	.	.	.
Individual level	**+**	**+**	**+**	**+**	**+**	**+**
Department level	**+**	**+**	**÷**	**÷**	**+**	**+**
**Support from immediate superior**	.	.	.	.	.	.
Individual level	**+**	**+**	**+**	**+**	**+**	**+**
Department level	**+**	**+**	**÷**	**÷**	**+**	**+**
**Fair leadership**	.	.	.	.	.	.
Individual level	**+**	**+**	**+**	**+**	**+**	**+**
Department level	**+**	**+**	**÷**	**÷**	**+**	**+**
**Predictability during the next month**	.	.	.	.	.	.
Individual level	**+**	**+**	**÷**	**÷**	**+**	**+**
Department level	**+**	**+**	**÷**	**÷**	**+**	**+**
**Commitment to organization**	.	.	.	.	.	.
Individual level	**+**	**+**	**+**	**+**	**+**	**+**
Department level	**+**	**+**	**÷**	**÷**	**+**	**+**
**Rumors of change**	.	.	.	.	.	.
Individual level	**+**	**+**	**+**	**÷**	**+**	**+**
Department level	**+**	**+**	**÷**	**÷**	**+**	**+**
**Human resource primacy**	.	.	.	.	.	.
Individual level	**+**	**+**	**+**	**+**	**+**	**+**
Department level	**+**	**+**	**÷**	**÷**	**+**	**+**
**Social climate**	.	.	.	.	.	.
Individual level	**+**	**+**	**+**	**+**	**+**	**+**
Department level	**+**	**+**	**÷**	**÷**	**+**	**+**

^a^Separate regressions were run for each factor.

^b^Age, sex, and skill level were included in all regressions.

^c^Age, sex, skill level, and outcome at baseline (T1) were included in all regressions.

#### Mental distress

The multilevel linear regression **“null-model”** with mental distress only exhibited statistically significant variance of 0.169 across *individuals* and of 0.004 across *departments* (Table 3). These variance components demonstrated that variation in mental distress was mainly between individuals rather than between departments. However, there was still statistically significant variation between departments.

Multivariable multilevel linear regressions of **baseline exposures** without baseline adjustment for mental distress demonstrated statistically significant relations between *all* work factors and mental distress two years after at both the *individual* and *department level*. *Role conflict* and *rumors of change* were associated with increased mental distress, while *decision control*, *positive challenge*, *fair leadership*, *predictability during the next month*, *support from immediate superior*, *commitment to organization*, *social climate*, and *human resource primacy* predicted lower levels of mental distress (Table 4).

Regression models with baseline exposures adjusted for baseline distress, demonstrated that *all* work factors were statistically significant predictors at the *individual level* except *predictability during the next month*. At the *department level*, none of the work factors were statistically significantly related to subsequent mental distress (Table 4).

Analyses of **average exposures** adjusted for mental distress at baseline exhibited statistically significant relations for *all* work factors with mental distress at both the *individual level* and the *department level*. *Role conflict* and *rumors of change* were associated with a higher level of mental distress, while *decision control*, *positive challenge*, *fair leadership*, *support from immediate superior*, *commitment to the organization*, *predictability during the next month*, *social climate*, and *human resource primacy* predicted a decrease in the level of mental distress (Table 4).

#### Positive affect

The multilevel **“null-model”** containing positive affect only demonstrated a statistically significant variance of 0.704 at the *individual level* and of 0.030 at the *department level* (Table 3). Compared to mental distress, the variance was larger at both levels. However, as for mental distress variation in positive affect was mainly at the individual level.

Analyses of **baseline exposures** with no adjustment for positive affect at baseline demonstrated that *all* work factors were statistically significant predictors of positive affect at follow-up, both at the *individual level* and *department level*. *Decision control*, *positive challenge*, *support from immediate superior*, *fair leadership*, *predictability during the next month*, *commitment to organization*, *human resource primacy*, and *social climate* were associated with higher levels of positive affect. *Role conflict* and *rumors of change* lowered the levels of positive affect (Table 5).

Multivariable multilevel regressions of baseline exposures including baseline positive affect revealed statistically significant fixed effects at the *individual level* for all work factors except for *predictability during the next month* and *rumors of change*. At the *department level*, none of the ten work factors statistically significantly predicted positive affect at follow-up (Table 5).

Analyses of **average exposures** adjusted for positive affect at baseline revealed statistically significant relations between *all* work factors and positive affect at both the *individual level* and the *department level*. *Decision control*, *positive challenge*, *fair leadership*, *support from immediate superior*, *commitment to the organization*, *predictability during the next month*, *social climate*, and *human resource primacy* predicted an increase in the level of positive affect while *role conflict* and *rumors of change* were associated with a decreased level (Table 5).

## Discussion

Elucidating relations between psychological work factors and “negative” and “positive” mental states should provide a practical basis for improving mental health and/or well-being in organizations. *All* ten work factors; *decision control*, *role conflict*, *positive challenge*, *support from immediate superior*, *fair leadership*, *predictability during the next month*, *commitment to organization*, *rumors of change*, *human resource primacy*, and *social climate* were related to mental distress *and* positive affect at both the *individual* and *department* level (Tables 4 and 5). However, analyses of baseline exposures adjusted for baseline outcome indicated statistically significant associations only at the *individual level*. Furthermore, baseline “rumors of change” was not related to baseline-adjusted positive affect and baseline “predictability during the next month” was not predictive of either outcomes when baseline outcome was adjusted for.

In recent years some studies have included aggregated measures of psychological work factors (e.g. [29, 30, 56, 57–59]). These *prospective* studies have mainly investigated relations between “classical” dimensions (e.g. “job demands”, “job control”, “effort-reward imbalance”) (e.g. [29, 58]) or “organizational justice” (e.g. [30, 56]), and “negative” outcomes. In accordance with Christensen et al [59], we found *decision control* and *positive challenge* (resembles “skill discretion”) to be department-level predictors of a “negative” outcome. Also, as in the current study, “leadership” at both levels and “influence at work” (resembles “decision control”) at the individual level have been related to a “positive” outcome [60].

Some cross-sectional multilevel studies, mainly investigating dimensions from Karasek`s DC model [21], have included both “negative” and “positive” outcomes [61, 62]. Overall, workplace characteristics, particularly at the aggregated level, were related to “positive” outcomes only [61, 62]. This is contrary to present results where most work factors were associated with both mental distress and positive affect at both levels. However, in accordance with our study, *decision control* was a predictor of “positive” outcomes at both the individual level and work unit level [62].

Psychological and social work factors were generally related to mental distress and positive affect in a mirrored way at both levels (Tables 4 and 5). This may indicate that mental distress and positive affect as currently measured represents opposite ends of a single dimension rather than being independent states. However, correlation coefficients of -0.51 at T1 and -0.53 at T2 showed some degree of independency. While depression has exhibited negative associations with positive affectivity as a trait, studies have reported no relationship of positive affectivity with anxiety (e.g. [63]). The present measure of mental distress (HSCL-10) captures symptoms of depression and anxiety [18, 19, 34], and has been suggested to be a measure of general psychological distress more than reflecting discriminate subscales of depression and anxiety [34, 64]. Hence, the possibility remains that different work factors influence positive affect than anxiety specifically.

Even though mental distress and positive affect shared most predictors, there were some differences. *Rumors of change* was associated with mental distress only across all designs, but not with positive affect when baseline exposure was adjusted for baseline-outcome. This factor has been found to predict depression [65] and mental distress “caseness” [23] in individual-level analyses. Another interesting difference was in relation to *predictability during the next month* (predictability of tasks, coworkers, and superiors) that exhibited a stronger influence on positive affect than on distress. This was the only work factor displaying strongest regression coefficients at the department level (see Tables 4 and 5), indicating a “contextual” effect, i.e. a group effect that does not seem fully mediated by employees`experience of working conditions [50]. Although most influential at the individual level, current results adds to the knowledge of “rumors of change” and “predictability during the next month” as factors that organizations should be aware of and target at both levels.

At both the individual level and department level associations were generally stronger for regressions with *positive affect* (Tables 4 and 5). This is in accordance with earlier cross-sectional multilevel studies [61, 62]. Hence, conditions in the workplace may have more impact on employees`positive emotions than on symptoms of anxiety and depression. However, differences related to the measurement instruments should be considered. The scale measuring positive affect assesses “normal” affect while the instrument measuring mental distress may capture clinical symptoms to a larger degree than assessing common distress. Positive affect is more prevalent than symptoms of anxiety and depression, and mental distress exhibited more stability over time.

In line with previous studies [61, 62], positive affect exhibited more variation between departments (see Table 3), and thereby seems more dependent on the group (i.e. the average worker) while symptoms of anxiety and depression is perhaps more dependent on individual idiosyncrasies’. Furthermore, mental distress carries more stigma and thereby may be kept more private than positive affect. Hence, positive affect at work is possibly more “contagious” and likely to spread throughout a work group [66] than mental distress. “Positive” outcomes like positive affect, engagement, job motivation, and job satisfaction may be better suited for studying at higher levels in organizations than health afflictions.

The basic assumption behind many “psychosocial work environment” dimensions is that they are exposures acting “independently” of the individuals’ psychological constitution. Intraclass-correlations of the current study (see “Results”) and in earlier studies (e.g. [29, 30, 56, 57–59]) demonstrating variation in work factors between departments support the notion of psychological and social work factors being, at least to some degree, shared among individuals [17, 50]. Hence, our findings imply that the work factors studied not only reflects individual, subjective experiences, but are also grounded in some kind of external, collective, and intersubjective “reality” [17, 50].

Our findings support a previously expressed notion that the contribution of psychological and social exposures is most pervasive at the individual level [67]. Individual evaluation or perceptions and appraisals of “external” factors are important intervening processes between the environment and individual response [68]. The individual appraisal process is characterized as a *moderator* and not a *confounder* inducing spurious associations. Hence, individual appraisals and traits may be important mechanisms on the pathway between work factors and mental health [69]. Support for negative affectivity being affected by working conditions and thereby acting as a *mediator* in the “stressor-strain” relationship has also been disclosed. Furthermore, positive affectivity has been found to act as a buffer [70].

Hence, the impact of psychological work factors may not be similar for all individuals in a work unit. Organizational interventions targeting individuals may consequently see the best results. However, relations at the department level were also discovered, indicating that improvement of working conditions at the work unit level, that is more practical and feasible to execute, will be a good alternative.

Challenges associated with aggregated measures may disguise relations at the department level. For instance, relations may not be detected due to restricted variation and insufficient exposure contrast. Limited variation between work units may be a problem as it diminishes statistical power [71]. Exposure misclassification or measurement imprecision may occur if failing to identify the organizational level at which individuals have a sufficient amount of shared work environment (see also [72, 73]). In the current study, aggregated variables were constructed on the basis of department information for each employee provided by the organizations. This provides information about formal functional work units. However, in some cases it may be more relevant to aggregate work factors on the basis of informal groups (see also [57]). Formal work units may be characterized by a high degree of heterogeneity in terms of each individual`s work content and responsibilities. Aggregating work factors to higher levels in organizations may be more appropriate in homogeneous units (see also [72]). Also, where some work factors (e.g. *social climate*, *human resource primacy*, *rumors of change*) seem well-suited for aggregation at the work unit, others (e.g. *decision control*, *role conflict*, *positive challenge*) may be better suited for aggregation at the job title (or other dimensions) (see also [72]). Current ICCs may reflect this (see “Results”). For instance, while ICC1 at baseline was 0.28 for human resource primacy, it was 0.11 for role conflict. Future multilevel studies should carefully consider which work factors should be aggregated at which dimensions.

A larger number of the associations, particularly at the department level, were statistically significant in the average-exposure design compared to in baseline-exposure models adjusted for baseline outcome. Furthermore, associations were stronger at both levels in models with average exposure. These results may have several explanations; i) long-term exposure is more likely to influence mental health, or ii) many work factors have short-term effects [74]. Furthermore, the cross-sectional element resulting from average exposure measures including exposure at T2 should be taken into account when interpreting the results. For a more in-depth discussion of this see [23]. The possibility of reversed causality “issues” should, however, be partly circumvented at the department level as aggregation should attenuate problems associated with possible reporting bias.

### Methodological considerations

The baseline response rate for individuals invited to the first survey was 53.3%. The attrition rate from baseline to follow-up was 32.3%. *External validity* may be affected if non-participants differ from participants. Differences were discovered in age (non-response and attrition), sex, skill level, mental distress, positive affect, and work factors (attrition) (see Table 2 and “Results”). Although the current study included a diverse sample, the *exact* population to which generalisation is valid cannot be accounted for *a priori* since the invited employees were not a randomly drawn representative population of the Norwegian (or any other country`s) working population. Hence, this selection has limited consequence for the external validity of the study. *Internal validity* may be threatened if self-selection is related to both exposures and outcome [75]. Current attrition analyses showed that some exposures (analyses not shown) and both outcomes (see Table 2) were associated with response at follow-up.

This selective dropout may have influenced results, e.g. by *healthy worker bias*. However, exposure-distress associations at baseline were similar for those who did and did not drop out, and no factors were statistically significant just for those responding only at T1 (analyses not shown). For *positive affect*, associations were somewhat stronger for those responding at *both* T1 and T2 for *decision control*, *positive challenge*, *role conflict*, *support from immediate superior*, *empowering leadership*, *fair leadership*, *predictability for the next month*, *commitment to the organization*, *rumors of change*, *procedural injustice*, and *human resource primacy*. However, no work factor was statistically significant only for those responding at both baseline and follow-up (analyses not shown). Although far from conclusive, this may suggest that repeated responders are more sensitive than dropouts to “positive effects” of work but not less sensitive to “negative effects”.

Psychological work factors (exposures), mental distress and positive affect (outcomes) were measured at the individual level by self-report. Possibly, correlated measurement errors (*common method bias*, CMB) may have inflated associations [76]. However, potential influences of CMB should be limited by the prospective design with temporal separation of measurements (e.g. situational factors inducing negative or positive states are not likely to occur at both baseline and follow-up) [76]. Also, the way QPS _Nordic_ is constructed should attenuate reporting biases (for further discussion of this see [23]) [8, 76]. Adjusting for mental distress and positive affect at baseline should attenuate CMB [76]. Aggregating work factors to the department level should also attenuate possible problems associated with reporting bias due to individual employee characteristics. Furthermore, the role of negative affectivity as a confounder between exposures at work and health outcomes has been questioned [31]. “Life stressors” (e.g. marital problems, illness in family members or close friends, financial problems) may induce spurious relations between work and mental health. For instance, an individual experiencing mental distress due to problems at home may attribute the symptoms to the work arena and thereby, for instance, rate his/hers superior as low on “fair leadership”. However, studies taking “life stressors” into account have identified an independent effect of conditions at work on mental health problems [77–79]. Also, prospective studies on a large cohort of Swedish twins taking potential confounding of “familial factors” (genetic and shared environment) into account have demonstrated independent effects of work factors on sick leave due to mental disorders [80], disability pension due to mental diagnoses [81], and burnout [82].

Regression models with baseline exposure without adjustment for baseline outcome demonstrated stronger coefficients, and that *all* exposures were associated with mental distress *and* positive affect at *both* the individual level and department level (see Tables 4 and 5). Differences between models with and without baseline adjustment were most significant at the department level. Adjustments for baseline outcome protect against type I errors, eliminating possible confounding if reversed effects between baseline outcome and baseline exposure is present [53]. However, type II errors may occur if mental distress/positive affect reported at baseline was influenced by previous or baseline exposure [53]. If so, adjusting for baseline outcome may constitute over adjustment eliminating possible effects of work factors (see [53, 54]), particularly if effects are short-term. Hence, the effect of baseline adjustment depends upon how the variables are causally related [53]. However, determining this within the framework of the current study with only two data waves is not possible.

### Concluding remarks

Overall, psychological and social work factors currently investigated were related in a mirrored way to *mental distress* and *positive affect*. Influence of workplace conditions seemed most extensive at the individual level, indicating subjective perceptions and experiences of work factors to be important in determining health effects. However, relations at the department level were also discovered. This suggests that the work factors, at least to some degree, also reflect something “objective” and external that is shared between employees, acting “independently” of individual idiosyncrasies’. Individual-level measures and aggregated work-unit measures may, thus, offer different perspectives on psychological work factors that act together in influencing employees`health and well-being. Organizational improvement efforts should be able to reduce distress *and* enhance positive affect by focusing on the specific work factors in the current study. Furthermore, interventions should target both individual employees *and* redesign of working conditions at the work unit level.

## Supporting Information

S1 Table Ordinary least square regression models with psychological and social work factors at baseline as predictors of mental distress at follow-up. (DOCX)

S2 Table Ordinary least square regression models with psychological and social work factors at baseline as predictors of positive affect at follow-up. (DOCX)

S3 Table Random components of multilevel linear regression models with psychological and social work factors at baseline and averaged across time ([T1+T2]/2) as predictors of mental distress at follow-up. (DOCX)

S4 Table Random components of multilevel linear regression models with psychological and social work factors at baseline and averaged across time ([T1+T2]/2) as predictors of positive affect at follow-up. (DOCX)
