# Supplementary material for: Psychological and Social Work Factors as Predictors of Mental Distress and Positive Affect: A Prospective, Multilevel Study
Source: PLoS One. 2016 Mar 24;11(3):e0152220. doi: 10.1371/journal.pone.0152220 (PMC4807036; doi:10.1371/journal.pone.0152220)
Supplement: S1 Table — (DOCX) [file pone.0152220.s001.docx]

| **S1 Table**. Ordinary least square regression models with psychological and social work factors at baseline as predictors of mental distress at follow-up^a b^. | **No adjustment for baseline dispositional optimism** | | | | | **Adjusted for baseline dispositional**  **optimism** | | | | |
| --- | --- | --- | --- | --- | --- | --- | --- | --- | --- | --- |
| **Exposure** | **N** | **B** | **β** | **99% CI** | **P-value** | **N** | **B** | **β** | **99% CI** | **P-value** |
| **Quantitative demands** | 3993 | 0.00 | 0.01 | -0.01- 0.02 | 0.579 | 3929 | 0.01 | 0.01 | -0.01- 0.02 | 0.400 |
| **Decision control** | 3975 | **-0.02** | **-0.04** | **-0.04 - -0.01** | **0.000** | 3913 | **-0.02** | **-0.04** | **-0.04- 0.00** | **0.005** |
| **Positive challenge** | 3814 | **-0.04** | **-0.07** | **-0.06- -0.02** | **0.000** | 3754 | **-0.03** | **-0.06** | **-0.05- -0.02** | **0.000** |
| **Role conflict** | 3987 | **0.03** | **0.06** | **0.02- 0.05** | **0.000** | 3924 | **0.03** | **0.06** | **0.02- 0.05** | **0.000** |
| **Support from immediate superior** | 3992 | **-0.03** | **-0.07** | **-0.04- -0.02** | **0.000** | 3929 | **-0.03** | **-0.06** | **-0.04- -0.01** | **0.000** |
| **Empowering leadership** | 3985 | **-0.02** | **-0.04** | **-0.03- 0.00** | **0.001** | 3923 | -0.01 | -0.03 | -0.03- 0.00 | 0.010 |
| **Fair leadership** | 3967 | **-0.02** | **-0.05** | **-0.04- -0.01** | **0.000** | 3905 | **-0.02** | **-0.04** | **-0.04- -0.01** | **0.000** |
| **Predictability during the next month** | 3994 | -0.01 | -0.02 | -0.03 -0.01 | 0.125 | 3932 | -0.01 | -0.02 | -0.03- 0.01 | 0.124 |
| **Predictability during the next two years** | 3666 | -0.01 | -0.03 | -0.02- 0.00 | 0.011 | 3613 | -0.01 | -0.02 | -0.02- 0.00 | 0.057 |
| **Rumors of change** | 3959 | **0.01** | **0.03** | **0.00- 0.02** | **0.008** | 3899 | 0.01 | 0.03 | 0.00- 0.02 | 0.013 |
| **Organizational procedural injustice** | 3645 | 0.01 | 0.01 | -0.01- 0.02 | 0.237 | 3596 | 0.00 | 0.01 | -0.01- 0.02 | 0.407 |
| **Commitment to organization** | 3895 | -0.01 | -0.03 | -0.03- 0.00 | 0.031 | 3840 | -0.01 | -0.02 | -0.02- 0.01 | 0.089 |
| **Human resource primacy** | 3830 | **-0.02** | **-0.05** | **-0.04- -0.01** | **0.000** | 3771 | **-0.02** | **-0.04** | **-0.04- -0.01** | **0.001** |
| **Social climate** | 3959 | **-0.03** | **-0.05** | **-0.05 - -0.01** | **0.000** | 3897 | **-0.03** | **-0.05** | **-0.05- -0.01** | **0.000** |

^a^Separate regressions were run for each factor.

^b^Age, sex, skill level, and mental distress at baseline (T1) were included in all regressions.
