# Supplementary material for: Psychological and Social Work Factors as Predictors of Mental Distress and Positive Affect: A Prospective, Multilevel Study
Source: PLoS One. 2016 Mar 24;11(3):e0152220. doi: 10.1371/journal.pone.0152220 (PMC4807036; doi:10.1371/journal.pone.0152220)
Supplement: S2 Table — (DOCX) [file pone.0152220.s002.docx]

| **S2 Table**. Ordinary least square regression models with psychological and social work factors at baseline as predictors of positive affect at follow-up^a b^. | **No adjustment for baseline dispositional optimism** | | | | | **Adjusted for baseline dispositional**  **optimism** | | | | |
| --- | --- | --- | --- | --- | --- | --- | --- | --- | --- | --- |
| **Exposure** | **N** | **B** | **β** | **99% CI** | **P-value** | **N** | **B** | **β** | **99% CI** | **P-value** |
| **Quantitative demands** | 3325 | 0.01 | 0.01 | -0.03- 0.05 | 0.473 | 3270 | 0.01 | 0.01 | -0.03- 0.05 | 0.526 |
| **Decision control** | 3309 | **0.10** | **0.10** | **0.06- 0.14** | **0.000** | 3255 | **0.08** | **0.08** | **0.04- 0.12** | **0.000** |
| **Positive challenge** | 3190 | **0.19** | **0.17** | **0.14- 0.23** | **0.000** | 3137 | **0.17** | **0.16** | **0.12- 0.21** | **0.000** |
| **Role conflict** | 3319 | **-0.06** | **-0.06** | **-0.10- -0.02** | **0.000** | 3264 | **-0.05** | **-0.05** | **-0.09- -0.01** | **0.001** |
| **Support from immediate superior** | 3326 | **0.09** | **0.10** | **0.06- 0.12** | **0.000** | 3270 | **0.08** | **0.09** | **0.04- 0.11** | **0.000** |
| **Empowering leadership** | 3319 | **0.09** | **0.12** | **0.06- 0.12** | **0.000** | 3265 | **0.08** | **0.10** | **0.05-0.11** | **0.000** |
| **Fair leadership** | 3304 | **0.08** | **0.09** | **0.05- 0.12** | **0.000** | 3250 | **0.07** | **0.08** | **0.03- 0.11** | **0.000** |
| **Predictability during the next month** | 3325 | **0.05** | **0.04** | **0.00- 0.09** | **0.005** | 3271 | 0.04 | 0.03 | -0.01- 0.08 | 0.023 |
| **Predictability during the next two years** | 3069 | **0.07** | **0.10** | **0.04- 0.09** | **0.000** | 3020 | **0.06** | **0.08** | **0.03- 0.08** | **0.000** |
| **Rumors of change** | 3302 | -0.01 | -0.02 | -0.04- 0.01 | 0.140 | 3249 | -0.01 | -0.01 | -0.03- 0.02 | 0.347 |
| **Organizational procedural injustice** | 3095 | **-0.06** | **-0.08** | **-0.09- -0.03** | **0.000** | 3049 | **-0.05** | **-0.07** | **-0.08- -0.02** | **0.000** |
| **Commitment to organization** | 3254 | **0.08** | **0.09** | **0.05- 0.12** | **0.000** | 3205 | **0.07** | **0.08** | **0.03- 0.10** | **0.000** |
| **Human resource primacy** | 3212 | **0.08** | **0.09** | **0.04- 0.11** | **0.000** | 3162 | **0.06** | **0.07** | **0.02- 0.09** | **0.000** |
| **Social climate** | 3300 | **0.09** | **0.08** | **0.05- 0.13** | **0.000** | 3246 | **0.07** | **0.06** | **0.03- 0.11** | **0.000** |

^a^Separate regressions were run for each factor.

^b^Age, sex, skill level, and positive affect at baseline (T1) were included in all regressions.
