# Supplementary material for: Psychological and Social Work Factors as Predictors of Mental Distress and Positive Affect: A Prospective, Multilevel Study
Source: PLoS One. 2016 Mar 24;11(3):e0152220. doi: 10.1371/journal.pone.0152220 (PMC4807036; doi:10.1371/journal.pone.0152220)
Supplement: S3 Table — (DOCX) [file pone.0152220.s003.docx]

| **Exposure** | | **Baseline exposure as predictor** | | | | | | | | **Average exposure as predictor^c^** | | | |
| --- | --- | --- | --- | --- | --- | --- | --- | --- | --- | --- | --- | --- | --- |
|  | | **No adjustment for baseline**  **mental distress^b^** | | | | **Adjusted for baseline**  **mental distress^c^** | | | |  | | | |
|  | | **N** | **Var.**  **comp** | ***SE*** | **P-value** | **N** | **Var.**  **comp** | ***SE*** | **P-value** | **N** | **Var.**  **comp** | ***SE*** | **P-value** |
| **Decision control** | | 4262^e^ | . | . | . | 3978^d^ | . | . | . | 3966^e^ | . | . | . |
|  | Ind. level intercept variance (r_ij_) | . | **0.158** | **0.006** | **0.000** | . | **0.096** | **0.004** | **0.000** | . | **0.092** | **0.004** | **0.000** |
|  | Dep. level intercept variance (u_0j_) | . | **0.006** | **0.002** | **0.003** | . | 0.002 | 0.001 | 0.041 | . | 0.002 | 0.001 | 0.023 |
|  | Slope variance (u_ij_) | . | 0.012 | 0.005 | 0.027 | . | . | . | . | . | 0.008 | 0.003 | 0.013 |
| **Role conflict** | | 4281^d^ | . | . | . | 3991^d^ | . | . | . | 3982^d^ | . | . | . |
|  | Ind. level intercept variance (r_ij_) | . | **0.158** | **0.006** | **0.000** | . | **0.095** | **0.004** | **0.000** | . | **0.093** | **0.004** | **0.000** |
|  | Dep. level intercept variance (u_0j_) | . | **0.005** | **0.002** | **0.007** | . | 0.002 | 0.001 | 0.042 | . | 0.002 | 0.001 | 0.046 |
|  | Slope variance (u_ij_) | . | . | . | . | . | . | . | . | . | . | . | . |
| **Positive challenge** | | 4086^e^ | . | . | . | 3815^e^ | . | . | . | 3683^e^ | . | . | . |
|  | Ind. level intercept variance (r_ij_) | . | **0.152** | **0.006** | **0.000** | . | **0.090** | **0.004** | **0.000** | . | **0.090** | **0.004** | **0.000** |
|  | Dep. level intercept variance (u_0j_) | . | **0.005** | **0.002** | **0.005** | . | 0.002 | 0.001 | 0.039 | . | 0.002 | 0.001 | 0.048 |
|  | Slope variance (u_ij_) | . | **0.021** | **0.006** | **0.001** | . | 0.008 | 0.004 | 0.025 | . | 0.008 | 0.004 | 0.055 |
| **Support from immediate superior** | | 4266^e^ | . | . | . | 3997^e^ | . | . | . | 3985^e^ | . | . | . |
|  | Ind. level intercept variance (r_ij_) | . | **0.149** | **0.006** | **0.000** | . | **0.091** | **0.004** | **0.000** | . | **0.089** | **0.004** | **0.000** |
|  | Dep. level intercept variance (u_0j_) | . | **0.006** | **0.002** | **0.002** | . | 0.002 | 0.001 | 0.011 | . | 0.002 | 0.001 | 0.017 |
|  | Slope variance (u_ij_) | . | 0.010 | 0.004 | 0.011 | . | 0.006 | 0.003 | 0.031 | . | 0.007 | 0.003 | 0.021 |
| **Fair leadership** | | 4225^e^ | . | . | . | 3970^e^ | . | . | . | 3947^e^ | . | . | . |
|  | Ind. level intercept variance (r_ij_) | . | **0.150** | **0.006** | **0.000** | . | **0.091** | **0.004** | **0.000** | . | **0.088** | **0.004** | **0.000** |
|  | Dep. level intercept variance (u_0j_) | . | **0.006** | **0.002** | **0.002** | . | **0.002** | **0.001** | **0.009** | . | 0.002 | 0.001 | 0.016 |
|  | Slope variance (u_ij_) | . | **0.013** | **0.005** | **0.004** | . | 0.009 | 0.004 | 0.015 | . | 0.009 | 0.004 | 0.017 |
| **Predictability during the next month** | | 4283^d^ | . | . | . | 3999^d^ | . | . | . | 3984^e^ | . | . | . |
|  | Ind. level intercept variance (r_ij_) | . | **0.165** | **0.006** | **0.000** | . | **0.096** | **0.004** | **0.000** | . | **0.093** | **0.004** | **0.000** |
|  | Dep. level intercept variance (u_0j_) | . | 0.005 | 0.002 | 0.014 | . | 0.002 | 0.001 | 0.040 | . | 0.002 | 0.001 | 0.049 |
|  | Slope variance (u_ij_) | . | . | . | . | . | . | . | . | . | 0.009 | 0.007 | 0.217 |
| **Commitment to organization** | | 4117^e^ | . | . | . | 3901^e^ | . | . | . | 3889^e^ | . | . | . |
|  | Ind. level intercept variance (r_ij_) | . | **0.153** | **0.006** | **0.000** | . | **0.092** | **0.004** | **0.000** | . | **0.090** | **0.004** | **0.000** |
|  | Dep. level intercept variance (u_0j_) | . | **0.005** | **0.002** | **0.006** | . | 0.002 | 0.001 | 0.018 | . | 0.002 | 0.001 | 0.020 |
|  | Slope variance (u_ij_) | . | 0.008 | 0.004 | 0.017 | . | 0.005 | 0.003 | 0.110 | . | 0.006 | 0.003 | 0.053 |
| **Rumors of change** | | 4244^e^ | . | . | . | 3962^d^ | . | . | . | 3924^d^ | . | . | . |
|  | Ind. level intercept variance (r_ij_) | . | **0.158** | **0.006** | **0.000** | . | **0.095** | **0.004** | **0.000** | . | **0.094** | **0.004** | **0.000** |
|  | Dep. level intercept variance (u_0j_) | . | 0.005 | 0.002 | 0.012 | . | 0.002 | 0.001 | 0.032 | . | 0.002 | 0.001 | 0.040 |
|  | Slope variance (u_ij_) | . | 0.004 | 0.002 | 0.041 | . | . | . | . | . | . | . | . |
| **Human resource primacy** | | 4041^d^ | . | . | . | 3833^d^ | . | . | . | 3706^e^ | . | . | . |
|  | Ind. level intercept variance (r_ij_) | . | **0.155** | **0.006** | **0.000** | . | **0.094** | **0.004** | **0.000** | . | **0.088** | **0.004** | **0.000** |
|  | Dep. level intercept variance (u_0j_) | . | 0.005 | 0.002 | 0.012 | . | 0.002 | 0.001 | 0.060 | . | 0.002 | 0.001 | 0.021 |
|  | Slope variance (u_ij_) | . | . | . | . | . | . | . | . | . | 0.007 | 0.003 | 0.047 |
| **Social climate** | | 4216^e^ | . | . | . | 3964^e^ | . | . | . | 3922^e^ | . | . | . |
|  | Ind. level intercept variance (r_ij_) | . | **0.150** | **0.006** | **0.000** | . | **0.090** | **0.004** | **0.000** | . | **0.089** | **0.004** | **0.000** |
|  | Dep. level intercept variance (u_0j_) | . | **0.005** | **0.002** | **0.005** | . | 0.002 | 0.001 | 0.013 | . | 0.002 | 0.001 | 0.054 |
|  | Slope variance (u_ij_) | . | **0.019** | **0.006** | **0.002** | . | **0.013** | **0.005** | **0.003** | . | 0.011 | 0.005 | 0.041 |

^a^Separate regressions were run for each factor.

**S3 Table.** Random components of multilevel linear regression models with psychological and social work factors at baseline and averaged across time ([T1+T2]/2) as predictors of mental distress at follow-up^a^.

^b^Age, sex, and skill level were included in all regressions.

^c^Age, sex, skill level, and mental distress at baseline (T1) were included in all regressions.

^d^Random intercept only model

^e^Random intercept and slope model
