# Supplementary material for: Psychological and Social Work Factors as Predictors of Mental Distress and Positive Affect: A Prospective, Multilevel Study
Source: PLoS One. 2016 Mar 24;11(3):e0152220. doi: 10.1371/journal.pone.0152220 (PMC4807036; doi:10.1371/journal.pone.0152220)
Supplement: S4 Table — (DOCX) [file pone.0152220.s004.docx]

| **Exposure** | | **Baseline exposure as predictor** | | | | | | | | **Average exposure as predictor^c^** | | | |
| --- | --- | --- | --- | --- | --- | --- | --- | --- | --- | --- | --- | --- | --- |
|  | | **No adjustment for baseline**  **positive affect^b^** | | | | **Adjusted for baseline**  **positive affect^c^** | | | |  | | | |
|  | | **N** | **Var.**  **comp** | ***SE*** | **P-value** | **N** | **Var.**  **comp** | ***SE*** | **P-value** | **N** | **Var.**  **comp** | ***SE*** | **P-value** |
| **Decision control** | | 3768^d^ | . | . | . | 3206^d^ | . | . | . | 3199^d^ | . | . | . |
|  | Ind. level intercept variance (r_ij_) | . | **0.664** | **0.017** | **0.000** | . | **0.482** | **0.015** | **0.000** | . | **0.478** | **0.015** | **0.000** |
|  | Dep. level intercept variance (u_0j_) | . | **0.031** | **0.008** | **0.000** | . | **0.015** | **0.006** | **0.008** | . | **0.015** | **0.006** | **0.006** |
|  | Slope variance (u_ij_) | . | . | . | . | . | . | . | . | . | . | . | . |
| **Role conflict** | | 3786^d^ | . | . | . | 3216^d^ | . | . | . | 3209^d^ | . | . | . |
|  | Ind. level intercept variance (r_ij_) | . | **0.662** | **0.017** | **0.000** | . | **0.481** | **0.015** | **0.000** | . | **0.478** | **0.015** | **0.000** |
|  | Dep. level intercept variance (u_0j_) | . | **0.031** | **0.008** | **0.000** | . | **0.014** | **0.005** | **0.009** | . | **0.015** | **0.005** | **0.007** |
|  | Slope variance (u_ij_) | . | . | . | . | . | . | . | . | . | . | . | . |
| **Positive challenge** | | 3618^d^ | . | . | . | 3092^d^ | . | . | . | 3015^d^ | . | . | . |
|  | Ind. level intercept variance (r_ij_) | . | **0.642** | **0.017** | **0.000** | . | **0.476** | **0.015** | **0.000** | . | **0.470** | **0.015** | **0.000** |
|  | Dep. level intercept variance (u_0j_) | . | **0.031** | **0.008** | **0.000** | . | **0.015** | **0.005** | **0.007** | . | 0.013 | 0.005 | 0.014 |
|  | Slope variance (u_ij_) | . | . | . | . | . | . | . | . | . | . | . | . |
| **Support from immediate superior** | | 3777^d^ | . | . | . | 3222^d^ | . | . | . | 3211^d^ | . | . | . |
|  | Ind. level intercept variance (r_ij_) | . | **0.648** | **0.017** | **0.000** | . | **0.479** | **0.015** | **0.000** | . | **0.468** | **0.014** | **0.000** |
|  | Dep. level intercept variance (u_0j_) | . | **0.028** | **0.007** | **0.000** | . | **0.014** | **0.005** | **0.008** | . | **0.014** | **0.005** | **0.007** |
|  | Slope variance (u_ij_) | . | . | . | . | . | . | . | . | . | . | . | . |
| **Fair leadership** | | 3737^d^ | . | . | . | 3201^e^ | . | . | . | 3187^d^ | . | . | . |
|  | Ind. level intercept variance (r_ij_) | . | **0.657** | **0.017** | **0.000** | . | **0.469** | **0.015** | **0.000** | . | **0.469** | **0.015** | **0.000** |
|  | Dep. level intercept variance (u_0j_) | . | **0.028** | **0.008** | **0.000** | . | **0.016** | **0.005** | **0.003** | . | 0.013 | 0.005 | 0.010 |
|  | Slope variance (u_ij_) | . | . | . | . | . | 0.023 | 0.009 | 0.010 | . | . | . | . |
| **Predictability during the next month** | | 3787^d^ | . | . | . | 3222^d^ | . | . | . | 3208^d^ | . | . | . |
|  | Ind. level intercept variance (r_ij_) | . | **0.677** | **0.017** | **0.000** | . | **0.483** | **0.015** | **0.000** | . | **0.481** | **0.015** | **0.000** |
|  | Dep. level intercept variance (u_0j_) | . | **0.028** | **0.008** | **0.000** | . | 0.013 | 0.005 | 0.013 | . | 0.012 | 0.005 | 0.018 |
|  | Slope variance (u_ij_) | . | . | . | . | . | . | . | . | . | . | . | . |
| **Commitment to organization** | | 3669^e^ | . | . | . | 3165^d^ | . | . | . | 3157^d^ | . | . | . |
|  | Ind. level intercept variance (r_ij_) | . | **0.634** | **0.017** | **0.000** | . | **0.482** | **0.015** | **0.000** | . | **0.475** | **0.015** | **0.000** |
|  | Dep. level intercept variance (u_0j_) | . | **0.031** | **0.008** | **0.000** | . | **0.015** | **0.005** | **0.006** | . | **0.014** | **0.005** | **0.007** |
|  | Slope variance (u_ij_) | . | 0.027 | 0.011 | 0.014 | . | . | . | . | . | . | . | . |
| **Rumors of change** | | 3757^d^ | . | . | . | 3201^d^ | . | . | . | 3174^d^ | . | . | . |
|  | Ind. level intercept variance (r_ij_) | . | **0.673** | **0.017** | **0.000** | . | **0.483** | **0.015** | **0.000** | . | **0.482** | **0.015** | **0.000** |
|  | Dep. level intercept variance (u_0j_) | . | **0.027** | **0.008** | **0.000** | . | 0.013 | 0.005 | 0.013 | . | 0.011 | 0.005 | 0.023 |
|  | Slope variance (u_ij_) | . | . | . | . | . | . | . | . | . | . | . | . |
| **Human resource primacy** | | 3589^d^ | . | . | . | 3116^d^ | . | . | . | 3042^d^ | . | . | . |
|  | Ind. level intercept variance (r_ij_) | . | **0.645** | **0.017** | **0.000** | . | **0.478** | **0.015** | **0.000** | . | **0.468** | **0.015** | **0.000** |
|  | Dep. level intercept variance (u_0j_) | . | **0.028** | **0.008** | **0.000** | . | **0.016** | **0.005** | **0.004** | . | **0.016** | **0.006** | **0.003** |
|  | Slope variance (u_ij_) | . | . | . | . | . | . | . | . | . | . | . | . |
| **Social climate** | | 3732^d^ | . | . | . | 3197^d^ | . | . | . | 3166^d^ | . | . | . |
|  | Ind. level intercept variance (r_ij_) | . | **0.657** | **0.017** | **0.000** | . | **0.483** | **0.015** | **0.000** | . | **0.474** | **0.015** | **0.000** |
|  | Dep. level intercept variance (u_0j_) | . | **0.027** | **0.008** | **0.000** | . | 0.014 | 0.005 | 0.011 | . | 0.013 | 0.005 | 0.011 |
|  | Slope variance (u_ij_) | . | . | . | . | . | . | . | . | . | . | . | . |

**S4 Table.** Random components of multilevel linear regression models with psychological and social work factors at baseline and averaged across time ([T1+T2]/2) as predictors of positive affect at follow-up^a^.

^a^Separate regressions were run for each factor.

^b^Age, sex, and skill level were included in all regressions.

^c^Age, sex, skill level, and positive affect at baseline (T1) were included in all regressions.

^d^Random intercept only model

^e^Random intercept and slope model
